# Clinical evaluation of glutathione concentrations after consumption of milk containing different subtypes of β-casein: results from a randomized, cross-over clinical trial

**DOI:** 10.1186/s12937-016-0201-x

**Published:** 2016-09-29

**Authors:** Richard Deth, Andrew Clarke, Jiayi Ni, Malav Trivedi

**Affiliations:** 1 Department of Pharmaceutical Sciences, College of Pharmacy, Nova Southeastern University, Rm # 1382, Terry Building, Fort Lauderdale, FL 33317 USA; 2 The a2 Milk Company Limited, Auckland, New Zealand; 3 S.P.R.I.M. China (Shanghai) Consulting Co., Ltd., Shanghai, China

**Keywords:** β-casein, Dairy, Glutathione, Milk, Redox

## Abstract

**Abstract:**

This study reports the plasma glutathione concentrations in a double-blind, randomized, controlled, 2 × 2 cross-over study in which healthy participants consumed conventional milk (2 × 250 mL per day) containing both A1 and A2 types of β-casein, or milk containing only A2 type β-casein. Beta-casomorphin-7 (BCM-7), a peptide uniquely derived from the A1 type of β-casein, was previously reported to downregulate glutathione expression in human gut epithelial and neuronal cell lines by limiting cysteine uptake. The current human study demonstrates that consumption of milk containing only A2 β-casein was associated with a greater increase in plasma glutathione concentrations compared with the consumption of milk containing both β-casein types, and did not increase plasma BCM-7 concentrations compared with the washout diet in the study participants. Thus, milk containing only A2 β-casein and not A1 β-casein has the potential to promote the production of the antioxidant glutathione in humans.

**Clinical Trial Registration:**

ClinicalTrials.gov; identifier: NCT02406469

**Electronic supplementary material:**

The online version of this article (doi:10.1186/s12937-016-0201-x) contains supplementary material, which is available to authorized users.

## Introduction

Disturbances in glutathione (GSH) and redox homeostasis contribute to the pathophysiological processes leading to neurodegenerative diseases [1], pancreatitis [2], and diseases associated with abnormal cell differentiation [3]. Therefore, maintaining redox balance is important in the context of disease prevention. In a recent study, we showed that exposure to β-casomorphin-7 (BCM-7), a proline-rich opioid peptide derived from bovine β-casein, caused a decrease of intracellular GSH concentrations in cultured neuronal SH-SY5Y cells [4]. This reduction in GSH was driven by a reduction in cellular uptake of cysteine, the rate-limiting precursor for GSH synthesis. Exposure to BCM-7 also reduced the ratio of reduced GSH to oxidized GSH (glutathione disulfide) as a marker of redox status, and the ratio of *S*-adenosylmethionine to *S*-adenosylhomocysteine as a marker of cellular methylation capacity. These results indicate that BCM-7 is a potential modulator of GSH.

BCM-7 is a bioactive peptide with high affinity to μ-opioid receptors. It is uniquely produced by proteolysis of the A1 but not the A2 type of β-casein in cow’s milk by digestive enzymes [5, 6]. β-casein is present in cow’s milk at a concentration of approximately 10 mg/mL and exists as one of a number of variants that are classed as either A1 or A2 type based on the flanking carboxyl amino acid residue and subsequently the potential to yield BCM-7. Either or both of these β-casein types may be expressed in milk, depending on the cow’s genetic makeup. The differences in the products generated by the proteolytic digestion of the A1 and A2 types of β-casein are due to a point mutation at amino acid residue 67 in the 209-amino acid β-casein protein. A histidine at this position in the A1 type readily allows enzymatic hydrolysis, whereas a proline at this position in the A2 type sterically hinders proteolytic cleavage [5]. By contrast, proteolysis of the A2 type of β-casein yields BCM-9, an opioid peptide with a much lower affinity for opioid receptors [5] and has demonstrable and contrasting effects on cell function and growth to BCM-7 [7]. However, Cieslinska et al. reported that BCM-7 was also generated from homogenous milk containing A2 β-casein, albeit at a level about four-times lower than its generation from homogenous milk containing A1 β-casein [8].

Although a previous report from the European Food and Safety Authority [9] acknowledged the production of BCM-7 from milk containing A1 β-casein, no physiological effects were reported. However, emerging preclinical and clinical evidence indicates that BCM-7 produced from A1 β-casein can have physiological effects e.g. [10–14].

Results of in vitro studies may not be reliably extrapolated to physiological outcomes in animals and humans. Several factors might influence the systemic concentrations of BCM-7 in animal and human studies, including the amount of BCM-7 produced from A1 β-casein contained in a typical serving of milk, the half-life of BCM-7 in the gastrointestinal tract, degradation by brush border dipeptidyl peptidase-4 (DPP-4) and the rate of transport of BCM-7 across the gastrointestinal tract into the systemic or potentially lymphatic circulation. Furthermore, other milk metabolites, notably those derived from whey proteins, have been reported to influence GSH concentrations by promoting cysteine absorption and providing a substrate for GSH synthesis [15, 16]. Therefore, it is necessary to conduct animal and human studies to examine if the effects of BCM-7 on GSH concentrations observed in vitro are also apparent in vivo following the consumption of A1 type protein and potential exposure to BCM-7.

In a preliminary in vivo study using rabbits, it was observed that consumption of a diet containing the A1 type of β-casein was associated with decreased cysteine concentrations in the ileum and decreased GSH concentrations in the blood, liver, and brain compared with consumption of the A2 type (Additional file 1: Figure S1). These data indicate that, if BCM-7 is not absorbed into the systemic circulation, GSH downregulation may occur because of limited intestinal cysteine uptake from the small intestine. Thus, it was speculated that a reduction in cysteine absorption into the circulation, secondary to downregulation of cellular cysteine uptake, contributed to the reduction in systemic GSH concentrations, which was coupled with reduced GSH concentrations in the brain. While these in vitro *and* in vivo results were consistent, it is necessary to extend this investigation to clinical trials in order to determine if the observed effects of the A1 and A2 types of β-casein impart corresponding effects on circulating GSH concentrations in humans.

**Fig. 1:**
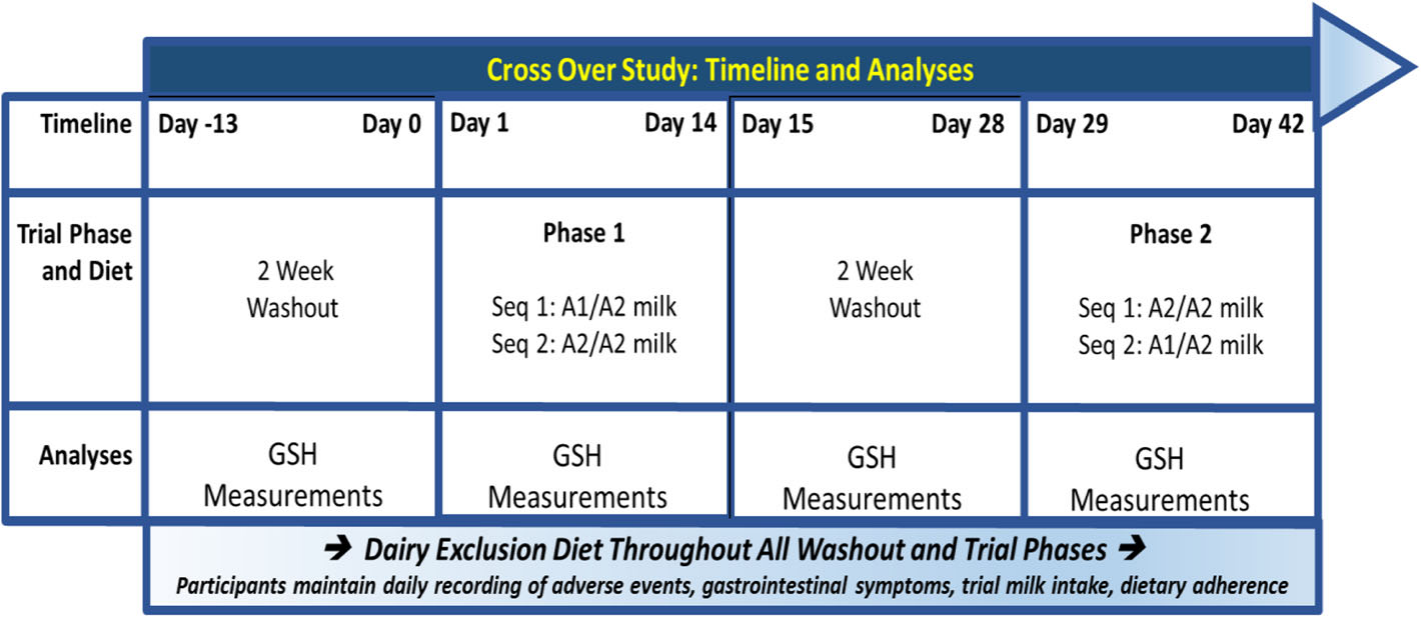
Study design. A1 = milk containing A1 and A2 β-casein; A2 = milk containing only A2 β-casein; GSH = glutathione. Modified from [9]

In consideration of these earlier observations from in vitro and in vivo studies, the effects of A1 and A2 types of β-casein on GSH concentrations were compared by collecting plasma samples from participants who consumed milk containing either the mixed A1/A2 type of β-casein or milk containing only the A2 type of β-casein in a recent double-blind, randomized, controlled, 2 × 2 cross-over study [12]. The design of the study is shown in Fig. 1. Plasma BCM-7 was also measured to determine if the systemic concentrations were differentially affected by the two diets, and if there was a correlation between the BCM-7 and GSH concentrations.

## Methods

### Study design

The design of the study is described in an earlier publication describing the effects of A1 and A2 β-casein consumption on aspects of digestive function, serum borne immune and inflammatory markers and cognitive function [12]. Because commercially available milk usually contains both A1 and A2 β-casein, we used a commercial milk product as the reference product, as well as milk containing only A2 β-casein, as described previously [12]. Eligible Chinese males or females included those aged 25–68 years who irregularly consumed milk, had self-reported mild to moderate digestive discomfort after milk consumption, and had normal electrocardiograms and blood pressure during quiet respiration. A total of 21 males and 24 females with a mean ± standard deviation age of 46.6 ± 14.0 years were enrolled. Twenty-three had confirmed lactase deficiency based on the results of urinary galactose tests.

The participants consumed commercially available conventional milk containing A1 and A2 types of β-casein (A1/A2) in phase 1 or commercially available milk containing only the A2 type of β-casein (A2) in phase 2 (A1/A2➔A2; sequence 1), or vice versa (A2➔A1/A2; sequence 2). The ratio of A1 to A2 β-casein in milk containing both types of β-casein was determined to be 42:58 by ultra-performance liquid chromatography method with diode array detection and tandem mass spectrometry. Each study phase lasted 2 weeks with 2-week washout periods before entering phase 1 and between phases 1 and 2. The participants were instructed to consume 250 mL of milk after 2 meals per day, every day. The subjects were prohibited from consuming other dairy products, but could consume non-dairy milk products during the study. The study was conducted in accordance with the Declaration of Helsinki as amended in Seoul 2008 and was approved by the ethics committee of the Shanghai Nutrition Society (approval number: SNSIRB#2014[002]). The study was registered with ClinicalTrials.gov (identifier: NCT02406469). All subjects provided written informed consent prior to inclusion in the study.

Blood samples were collected at baseline and at the end of each study phase for the measurement of a range of biomarkers, including GSH and BCM-7. Plasma was stored at −80 °C until required for assays. Several other biomarkers were measured and the results are published elsewhere [12].

### Measurement of total plasma GSH

Plasma samples were thawed in ice, and 5 μL of a 0.4 N perchloric acid solution was added to 200 μL of plasma to precipitate any remaining proteins. The total protein concentration was calculated and used to normalize GSH concentrations as previously described [17]. Total GSH concentrations were measured using a commercially available glutathione assay kit (Sigma-Aldrich, St. Louis, MO, USA). The kit involves catalytic conversion of GSH with 5,5′-dithio-2-nitrobenzoic acid and GSSG is recycled by glutathione reductase to release NADP+, resulting in the formation of two 5-thio-2-nitrobenzoic acid molecules that form a yellow color measured at 412 nm. The results were calculated based on the nmol of 5-thio-2-nitrobenzoic acid formed as min^−1^ mg^−1^ protein, which was converted to nmol/mg of protein. The GSH concentration was measured twice for each sample in independent assays.

### Measurement of plasma BCM-7 concentrations

BCM-7 concentrations were measured in blood samples obtained at 3 h after a meal. Plasma samples were treated with diprotin-B to inhibit DPP-4 activity. BCM-7 was measured by high performance liquid chromatography coupled to mass spectrometry (MS) at an external laboratory in the multiple reaction monitoring (MRM) mode (Pharmaceutical Analysis Center, School of Pharmacy, Second Military Medical University, Shanghai). The method used to measure BCM-7 is described in more detail in the Additional file 1.

### Statistical analysis

GSH concentrations were highly skewed based on the Kolmogorov–Smirnov test. Therefore, the GSH concentrations were analyzed using the Wilcoxon two-sample test with phase and cross-over as fixed effects, and *P* < 0.05 was taken to indicate a statistically significant phase or cross-over effect. Although BCM-7 concentrations were not skewed based on the Kolmogorov–Smirnov test, similar analyses were also performed for the BCM-7 concentrations.

## Results

**Fig. 2:**
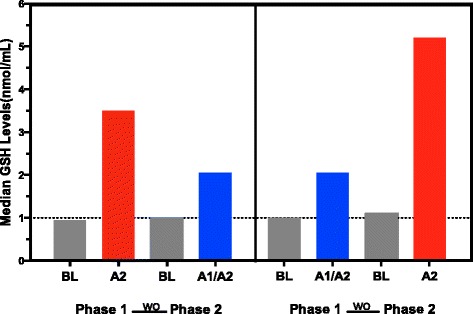
Median glutathione (GSH) concentrations before and after consumption of milk. Participants consumed milk containing both the A1 and A2 types, or only the A2 type of β-casein. Data are shown for the first of two repeated measures in each participant; the results of both measures were similar. The phase difference was statistically significant (*P* = 0.0059) but the cross-over difference was not significant (*P* = 0.7615) (Wilcoxon two-sample test). A1 = milk containing A1 and A2 β-casein; A2 = milk containing only A2 β-casein; BL = baseline; WO = washout

The median GSH concentrations measured at the start and end of each study phase in both sequences are depicted in Fig. 2. Results of the Wilcoxon two-sample test are shown in Additional file 1: Table S1. Consumption of milk containing only the A2 β-casein type was associated with significantly greater increases in plasma GSH concentrations from baseline to the end of the study phase compared with the consumption of milk containing both β-casein types. This increase occurred in both sequences irrespective of which milk product was consumed first. The mean ± standard error of the mean change in GSH concentrations from baseline was 4.01 ± 0.61 nmol/mL for milk containing A2 β-casein compared with 1.99 ± 0.50 nmol/mL for milk containing both types of β-casein. The change in GSH concentrations from baseline with the A2 type of β-casein tended to be greater in phase 1 (Sequence A2➔A1/A2) than in phase 2 (Sequence A1/A2➔A2) (4.07 vs. 2.70 nmol/mL).

Plasma BCM-7 concentrations were significantly greater in samples obtained during consumption of milk containing both β-casein types than at baseline during consumption of milk containing A2 β-casein (Additional file 1: Table S2). The BCM-7 concentrations were not significantly different between samples obtained after consumption of milk containing A2 β-casein and the washout/baseline phases.

## Discussion

The objective of the current analyses was to elucidate the effects of consuming milk containing the A1 or A2 types of β-casein on circulating GSH concentrations in humans, using blood samples obtained in a randomized 2 × 2 cross-over study [13]. The present results indicate that the consumption of milk containing only A2 β-casein was associated with greater increases in plasma GSH concentrations compared with the consumption of milk containing both β-casein types, and this was independent of a cross-over effect. The potential physiological benefits of elevated GSH concentrations are due to an elevated plasma antioxidant capacity, which allows aerobic metabolism to proceed without incurring damage from accumulating reactive oxygen species (ROS), while maintaining the homeostatic redox equilibrium [18]. Because GSH also plays a major role in detoxification, the two-fold higher plasma GSH concentration associated with the consumption of milk containing only A2 β-casein may provide greater protection against a range of environmental exposures. Reduced GSH concentrations are implicated in a range of diseases, including neurodegenerative, cardiovascular, pulmonary, immune, and inflammatory diseases, as well as cystic fibrosis. Hence, elevated GSH concentrations could be beneficial to patients with such diseases; however, any direct beneficial correlation between milk containing A2 β-casein and such diseases needs to be clinically investigated.

Although we did not measure cysteine concentrations in the current study, prior studies showed that whey protein, which does not yield BCM-7 during proteolysis, promotes cysteine absorption and the synthesis of GSH [15, 19–21]. By contrast, the opioid peptides derived from β-casein and wheat gluten protein inhibit cysteine uptake, decrease GSH concentrations and the anti-oxidant potential (i.e., decrease the GSH/glutathione disulfide ratio) [17], with subsequent epigenetic consequences [17, 22]. Although epigenetic changes were not assessed in the current study, such changes could contribute to the underlying elevated GSH concentrations in the second phase of the current study, especially because such epigenetic changes are sustained for a long time [17, 22]. Hence, the potential epigenetic effects of these opioid peptides warrant further clinical investigation.

The results obtained in this randomized cross-over study are consistent with the results of these prior studies. Eliminating the A1 type of β-casein from the milk diet allowed for greater increases in GSH synthesis, likely through eliminating the inhibitory effects of BCM-7 on cysteine uptake by the small intestine, as demonstrated in animal studies and in cells exposed to BCM-7 [17]. This is also supported by the lower BCM-7 concentrations observed in the A2 β-casein periods compared with the A1/A2 β-casein periods in the present study.

It is also notable that, even though BCM-7 concentrations were proportionately greater after consumption of milk containing both β-casein types than after consumption of milk containing the A2 type (Additional file 1: Table S2), the concentrations were lower than those reported to elicit biological effects in cultured human gut epithelial or neuronal cells [17]. Additionally, the concentrations were lower than those in individuals with neurological disorders or DPP-4 deficiency [23]. This observation is indicative that the measurements of plasma BCM-7 may be misleading, because BCM-7 transport and subsequent systemic tissue exposure might be influenced by alternative transport mechanisms. Thus, the chaotrophic environment of peptide extraction procedures seemingly rules out sequestering or compartmentalizing of BCM-7 by blood borne molecules or cells, and opens up the possibility of endocytosis or binding of macrophages and subsequent uptake by dendritic cells through the lymphatic system. This mechanism was proposed by Martin et al. to explain the transport of macromolecules from the small intestine to other tissues in the body [24], and the observation implies that BCM-7 concentrations should be measured in other tissue components, such as lymph tissue, in future studies.

Another notable finding is that BCM-7 was detected in baseline samples, which were obtained after a dairy washout phase. These findings indicate that A1 β-casein protein was not entirely eliminated from the diet, probably because dairy protein is present in non-milk foods, including processed foods, allowing the production of BCM-7 from dairy proteins or caseinates. Changes in the expression of DPP-4 could also influence the systemic BCM-7 concentrations.

These observations and prior findings support the proposition that BCM-7 may act by inhibiting cysteine absorption via gut epithelial cells, ultimately reducing the circulating GSH concentrations. Other dietary and physiological factors are also expected to influence the circulating GSH concentrations, and may explain the differences in GSH concentrations between the A2 β-casein phases. Although it was not possible to examine the direct impact of complete elimination of dietary BCM-7 on circulating GSH concentrations, the diet-dependent changes observed in the present study indicate that the residual BCM-7 concentrations detected in the A2 β-casein phase participants were comparable to the washout period without supplementary A1 β-casein consumption and did not adversely affect GSH uptake.

In conclusion, the results of this study suggest that daily consumption of commercially available conventional milk is associated with an increase in GSH concentrations, possibly as a consequence of increased supply of cysteine in whey protein. However, the magnitude of this increase in GSH concentrations was greater when the participants consumed milk containing the A2 type of β-casein as compared with milk containing both A1 and A2 β-casein. This elevation might be attenuated by the presence of the A1 type of β-casein relative to milk containing only the A2 type of β-casein. These results suggest that eliminating the A1 type of β-casein from milk may allow for a greater increase in GSH concentrations or maximize the potential for GSH production, and hence confer greater antioxidant capacity.

## Additional file

Additional file 1: Detailed Method to isolate and measure BCM-7. (DOC 189 kb)

## Abbreviations

BCM-7: β-casomorphin-7
DPP-4: Dipeptidyl peptidase-4
GSH: Glutathione
MRM: Multiple reaction monitoring
MS: Mass spectrometry
