# Supplementary material for: Clinical evaluation of glutathione concentrations after consumption of milk containing different subtypes of β-casein: results from a randomized, cross-over clinical trial
Source: Nutr J. 2016 Sep 29;15:82. doi: 10.1186/s12937-016-0201-x (PMC5041571; doi:10.1186/s12937-016-0201-x)
Supplement: Additional file 1: — Detailed Method to isolate and measure BCM-7. (DOC 189 kb) [file 12937_2016_201_MOESM1_ESM.doc]

**Additional file 1**

**Supplemental Methods:**

**Plasma samples used for method validation and standard curve generation**

A plasma sample was used to validate the BCM-7 MS analysis method. Six 50 µL aliquots (S1, S2, S3, S4, S5 and S6) of this plasma were spiked with 0, 0.5, 5, 25, 100 and 250 pg of synthetic BCM-7 (YPFPGPI, molecular weight 790.3 Da; Neo-Peptide (Boston, MA) and spiked with 1.5 ng of synthetic BCM-7* (stable isotope labelled BCM-7, YPF*PGPI, where phenylalanine had nine C13 and one N15, molecular weight 800.4 Da; Auspep, Tullamarine, Victoria, Australia). Therefore, the six samples contained the equivalent of 0, 0.01, 0.1, 0.5, 2 and 5 ng/mL of free BCM-7 and 30 ng/mL of free BCM-7* in 50 µL of plasma. Up to 50 µL of water was added to adjust the sample volumes so that all samples had the same volume. Then, 110 µL of 1% formic acid was added before peptide solid phase extraction.

**Solid phase extraction**

A C18 cartridge (Waters peptide extraction cartridge; Waters, Milford, MA, USA) was used for peptide extraction. The cartridge was first washed with 1 mL of buffer B (60% acetonitrile, 39% water, 1% trifluoroacetic acid), then equilibrated with 3 mL of buffer A (1% trifluoroacetic acid, 99% water) by gravity flow. After preparing the cartridge, the sample was loaded onto the cartridge, washed with 2 mL of buffer A and then eluted with 1 mL of buffer B by gravity flow. The collected eluent was dried in a vacuum concentrator (SpeedVac; Thermo Fisher Scientific, Waltham, MA, USA). All samples were reconstituted in 50 µL of 0.1% formic acid before MS data acquisition.

**Mass spectrometry**

Each sample (5 µL) was injected onto the peptide trap (Symmetry C18, 180 µm × 10 mm, 3.5 µm; Waters) for desalting and pre-concentrating with 0.1% formic acid, at a flow rate of 5 µL/mL. After 10 min, the peptide trap was switched in-line with the analytical column (BEH130C18, 1.7 µm, 100 µm × 100 mm; Waters). Peptides were eluted from the column using a linear solvent gradient, with steps from H2O:CH3CN (99:1; + 0.1% formic acid) to H2O:CH3CN (50:50; + 0.1% formic acid) at 400 nL/min over 30 min. The liquid chromatography eluent was subjected to nanoflow electrospray MRM analysis with a dwell time of 150 ms for BCM-7 and BCM-7* peptides, and the Y2, Y3 and Y4 ions. The MRM monitored ions were 790.4/229.1, 790.4/286.1, 790.4/383.2 for BCM-7 and were 800.4/229.1, 800.4/286.1, 800.4/383.2 for BCM-7*. A blank sample was injected to eliminate background interference before each sample injection.

**Multiple reaction monitoring data processing and results**

BCM-7* has identical chemical properties to BCM-7. It co-elutes with BCM-7 in nanoLC and has an identical product ion intensity pattern to BCM-7. Therefore, it is the ideal internal standard to identify and quantify BCM-7 in plasma samples. The MRM peak pattern and the peak retention time that matched BCM-7* was used to identify BCM-7 peaks. The MRM ion chromatogram peak areas were integrated using Multiquant (AB Sciex, Mt Waverley, Victoria, Australia). The BCM-7 concentrations for the samples were calculated using the following equation:

[BCM-7] = [BCM-7*] × A_BCM-7_ /A_BCM-7*_

Where [BCM-7] is the measured BCM-7 concentration, [BCM-7*] is the BCM-7* internal standard concentration, A_BCM-7_ is the BCM-7 extracted ion chromatogram peak area and A_BCM-7*_ is the BCM-7* extracted ion chromatogram peak area. Using this method, the [BCM-7] for samples S1, S2, S3, S4, S5 and S6 were measured and compared with the spiked [BCM-7].


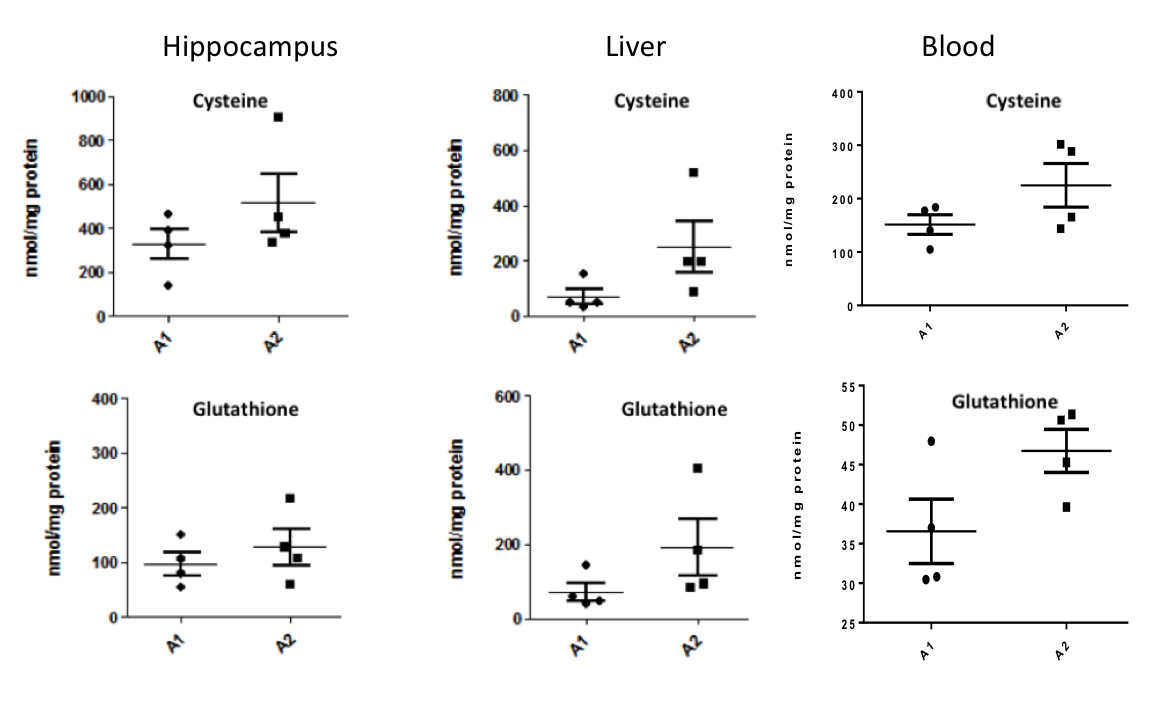


**Figure S1.** Cysteine and glutathione concentrations in the hippocampus, liver, and blood from rabbits.

Rabbits were fed a diet containing either the A1 and A2 types of β-casein or only the A2 type of β-casein. Results are shown as the mean ± standard error of the mean for 4 rabbits per group.

A1 = diet containing A1 and A2 β-casein; A2 = diet containing only A2 β-casein.

**Table S1.** Exact Wilcoxon two-sample test for plasma glutathione concentrations

|  |  | **Sequence** | **N** | **Wilcoxon rank sum** | **Expected under H0** | **Standard deviation under H0** | **Mean score** | ***P*-value** |
| --- | --- | --- | --- | --- | --- | --- | --- | --- |
| **First measure** | **Phase** | A1/A2🡪A2 | 22 | 386.5 | 506 | 44.0402 | 17.57 | 0.0059 |
|  |  | A2🡪A1/A2 | 23 | 648.5 | 529 | 44.0402 | 28.20 |  |
|  | **Cross-over** | A1/A2🡪A2 | 22 | 520.0 | 506 | 44.0416 | 23.64 | 0.7615 |
|  |  | A2🡪A1/A2 | 23 | 525.0 | 529 | 44.0416 | 22.39 |  |
| **Second measure** | **Phase** | A1/A2🡪A2 | 22 | 386.0 | 506 | 44.0416 | 17.55 | 0.0058 |
|  |  | A2🡪A1/A2 | 23 | 649.0 | 529 | 44.0416 | 28.22 |  |
|  | **Cross-over** | A1/A2🡪A2 | 22 | 517.0 | 506 | 44.0416 | 23.50 | 0.8134 |
|  |  | A2🡪A1/A2 | 23 | 518.0 | 529 | 44.0416 | 22.52 |  |

A1/A2 = milk containing A1 and A2 β-casein; A2 = milk containing only A2 β-casein.

**Table S2.** Plasma β-casomorphin-7 concentrations according to sequence and phase

| **Variable** | **Sequence 1^a^** | | | | **Sequence 2^b^** | | | | **Mixed-effects ANOVA** | | |
| --- | --- | --- | --- | --- | --- | --- | --- | --- | --- | --- | --- |
|  | **Phase 1** | | **Phase 2** | | **Phase 1** | | **Phase 2** | | **Estimate^c^** | **(SD)** | ***P*-value** |
|  | **BL** | **PI** | **BL** | **PI** | **BL** | **PI** | **BL** | **PI** |
| **BCM-7 (ng/mL)** | 0.63 | 0.976 | 0.71 | 0.71 | 0.56 | 0.73 | 0.65 | 0.87 | 0.03536 | 0.01723 | **0.0291^d^** |

^a^Sequence 1: A1/A2→A2

^b^Sequence 2: A2→A1/A2

^c^A1/A2 − A1.

^d^Statistically significant at *P* < 0.05.

A1 = milk containing A1 and A2 β-casein; A2 = milk containing only A2 β-casein; ANOVA = analysis of variance; BCM-7 = β-casomorphin-7; BL = baseline; PI = postintervention (i.e., after 2 weeks of each intervention); SD = standard deviation.
